# Supplementary material for: Nicotine Neurotoxicity Involves Low Wnt1 Signaling in Spinal Locomotor Networks of the Postnatal Rodent Spinal Cord
Source: Int J Mol Sci. 2021 Sep 3;22(17):9572. doi: 10.3390/ijms22179572 (PMC8431663; doi:10.3390/ijms22179572)
Supplement: Supplementary file 1 [file ijms-22-09572-s001.zip › ijms-1338937-supplementary.pdf]

International Journal of *Molecular Sciences*

MDPI

Article

Nicotine Neurotoxicity Involves Low Wnt1 Signaling in Spinal Locomotor Networks of the Postnatal Rodent Spinal Cord

Jaspreet Kaur [1,2,*], Graciela L. Mazzone [2,3,*], Jorge B. Aquino [3], and Andrea Nistri [2]

[1] Faculty of Health and Medical Sciences, Department of Neuroscience, University of Copenhagen, Blegdamsvej 3, DK-2200 KBH N, Denmark
[2] Department of Neuroscience, International School for Advanced Studies (SISSA), 34136 Trieste, Italy; nistri@sissa.it
[3] Instituto de Investigaciones en Medicina Traslacional (IIMT), CONICET-Universidad Austral, Av. Pte. Perón 1500, Pilar, B1629AHJ Buenos Aires, Argentina; jaquino@austral.edu.ar
* Correspondence: jaspreet.kaur@sund.ku.dk (J.K.); GMazzone@austral.edu.ar (G.L.M.); Tel.: +45-5260-1502 (J.K.); +54-23-0438-7425 (G.L.M.)

Supplemental Figure Legends

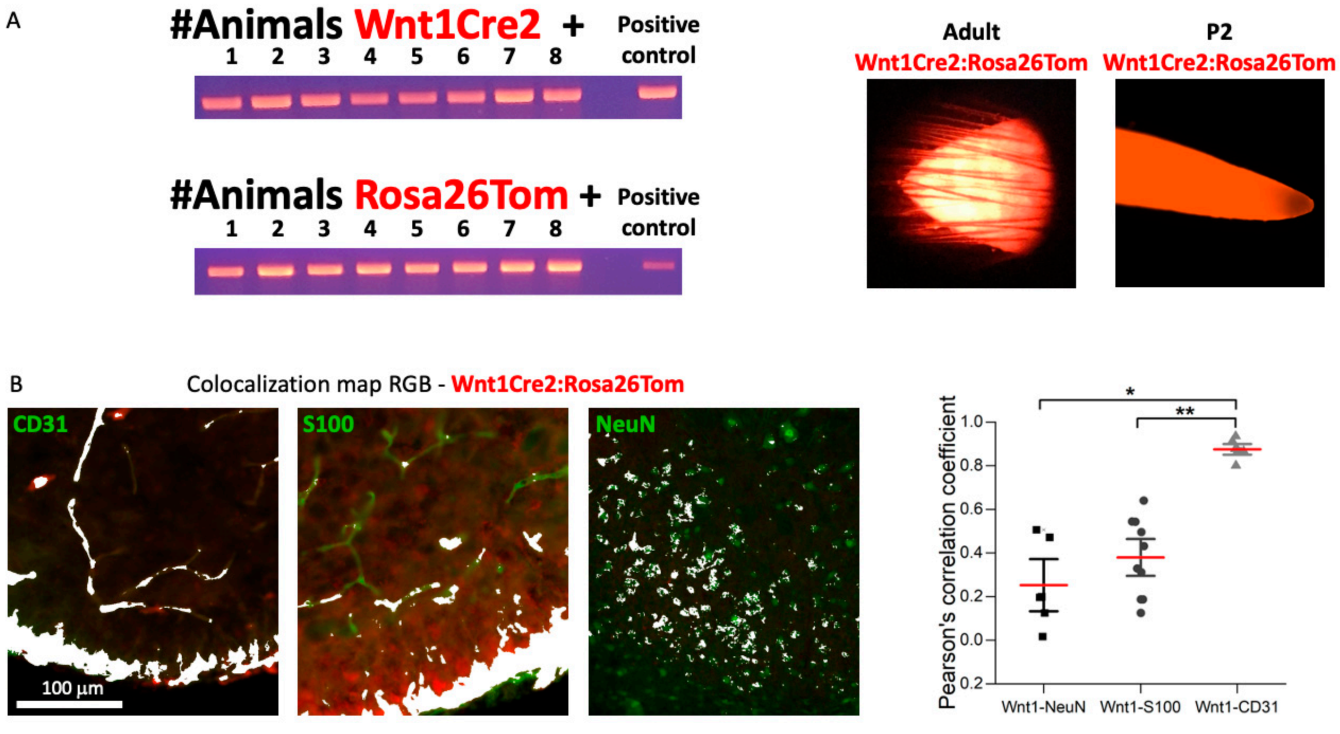

Figure S1. (A) Left: bands showing the expression of Cre and Tomato in Wnt1Cre2:Rosa26Tom mouse tail samples by PCR. Right: examples of Wnt1Cre2:Rosa26Tom tail tips of adult and postnatal (P2) mice. **(B)** Representative binary versions of images of Wnt1Cre2:Rosa26Tom signal with CD31, S100 or NeuN marker. After applying Costes threshold, pixels with positive signals for both are shown in white. Whisker plot showing a comparison of Pearson's Correlation Coefficient for CD31, S100 or NeuN and Wnt1Cre2:Rosa26Tom: 0.87 ± 0.01, 0.20 ± 0.07, 0.41 ± 0.05, respectively (S100 vs CD31: $^{**}p < 0.004$; NeuN vs CD31: $^{*}p < 0.047$; Dunn's Method). Scale bar (100 µm) applies to all panels. Note that mean bars in the whisker plots are represented in red.

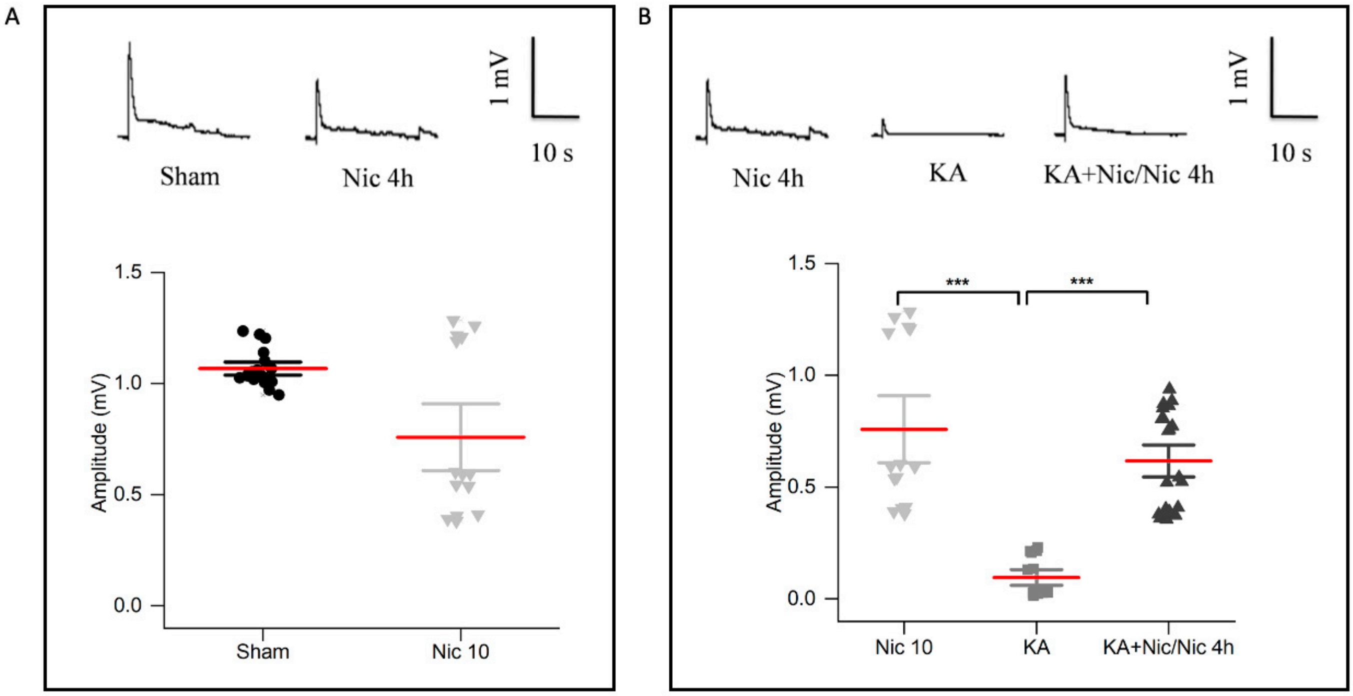

Figure S2. D-DRPs recorded from rat lumbar spinal cord. **(A)** Nic 10 ($n = 3$) application (4 h) did not block the amplitude in comparison to sham ($n = 4$) as indicated in the panel A example and histogram ($p = 0.106$, U = 83, Mann-Whitney test). **(B)** KA ($n = 4$) application drastically reduced the D-DRP amplitude (Nic 10 vs KA: ***$p \leq 0.001$, U = 0, Mann-Whitney test) which was recovered to normal after Nic 10 co-application ($n = 5$) (Nic 10 vs KA+Nic/Nic 4 h: ***$p \leq 0.001$, U = 0, Mann-Whitney; ***$p \leq 0.001$, $H_{(2)} = 30.156$, Kruskal-Wallis One Way Analysis of Variance on Ranks test). Note that mean bars in the whisker plots are represented in red.

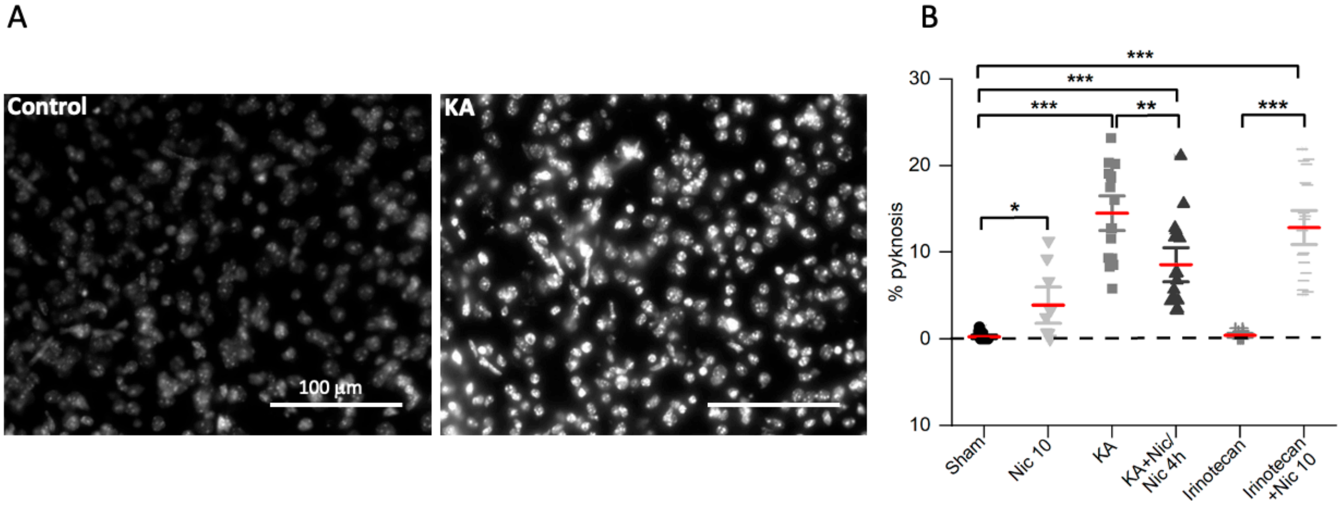

Figure S3. Pyknosis evoked by nicotine, KA or irinotecan in the ventral spinal region of mice. **(A)** Images showing nuclear staining by DAPI in sham and KA treated sections. **(B)** Whisker plot showing % pyknosis evoked by Nic 10 (sham vs Nic 10: *$p = 0.03$, $t_{8.15} = -2.616$, Welch's *t*-test), KA (sham vs KA: ***$p \leq 0.001$, $t_{15.31} = -10.629$, Welch's *t*-test) or KA+Nic/Nic 4h (KA vs KA+Nic/Nic 4h: **$p = 0.003$, $t_{29.975} = 3.195$, Welch's *t*-test) treatment. Extensive pyknosis was

evoked by irinotecan+Nic 10 treatment (sham vs Irinotecan+Nic 10: ***$p \leq 0.001$, $t_{18.4} = -9.58$; Irinotecan vs Irinotecan+Nic 10: ***$p \leq 0.001$, $t_{18.496} = -9.435$, Welch's *t*-test). Scale bar (100 μm) applies to all panels. Note that mean bars in the whisker plots are represented in red.
